# Murray on the Treatment of Cholera

**Published:** 1874-07

**Authors:** 


OBSERVATIONS ON THE PATHOLOGY AND TREATMENT OF CHOLERA. *The result of forty years experience.* By John Murray, M.D., Inspector General of Hospitals, late of Bengal. New York: G. P. Putnam's Sons, 1874. 12 mo., cloth., pp. 58. Price $1.00.

We find this little volume rich in the stores of long practical experience with the malady in question; an experience gained by its study in its natural habitat, India. The work is eminently practical in its character, being devoted chiefly to the treatment of the malady. The author is an advocate of elimination by purgatives.

---

MESSRS. WILLIAM WOOD & Co. announce that they propose to publish by *subscription* an ENCYCLOPÆDIA OF THE PRACTICE OF MEDICINE, translated from the German. It will be an entirely new work, from the pens of the more eminent German practitioners. It is proposed to issue the work in fifteen volumes, four of which will appear each year until completed. Terms of subscription, payable on delivery of each volume; in cloth, $5.00; in leather, $6.00; in half morocco, $7.50 per volume. Subscription may be made to the publishers, at 27 Great Jones street, New York.

---

INTRA UTERINE FIBROIDS. By J. Marion Sims, M.D., etc.: New York, 1874. pp. 27.

---

URETHROTOMY. By Fessenden N. Otis, M.D., etc.: New York, 1874. pp. 24.

---

SYPHILITIC MEMBRANOID OCCLUSION OF THE RIMA GLOTTIDIS. By Louis Elsberg, M.D., etc.: New York, 1874. pp. 16.

---

MUTUAL RELATIONS OF DRUGGISTS AND PPYSICIANS. By Charles E. Buckingham, M.D., etc.: Boston, 1874. pp. 15.

---

YELLOW FEVER IN MEMPHIS IN 1873. By R. W. Mitchell, M.D.: 1874. pp. 11.

CLIMATE IN PULMONARY CONSUMPTION, AND CALIFORNIA AS A HEALTH RESORT. By Lewis Rogers, M.D.: 1874. pp. 16.

---

CIVIL SERVICE OF THE UNITED STATES—REPORT TO THE PRESIDENT. Washington, 1874. pp. 98.

---

ELECTROLYSIS IN THE TREATMENT OF STRICTURE OF THE URETHRA. By Robert Newman, M.D., etc.: New York. T. S. Clacher, 1874. pp. 32.

---

THE TREATMENT OF UETRINE FLEXIONS. By Ely Van de Warker, M.D.: Syracuse, N. Y., 1874. pp. 13.

IS OVULATION THE SOLE CAUSE OF MENSTRUATION? By C. C. Matteson, M.D.: Philadelphia, 1874. pp. 7.

---

SPECIAL RULES FOR THE MANAGEMENT OF INFANTS DURING THE HOT SEASON, *recommended by the Obstetrical Society of Philadelphia:* 1874. pp. 8.

---

THE INDIAN SPRING, BUTTS COUNTY, GEORGIA—THE MINERAL WATER. *Historic and hygienic review, with analysis of the water.* Atlanta, Ga.: James P. Harrison & Co., 1874. pp. 26.

